# Challenges for a Local Service Agency to Address Domestic Violence –A Case Study From Rural Indonesia

**DOI:** 10.5539/gjhs.v6n6p214

**Published:** 2014-08-15

**Authors:** Elli Nur Hayati, Maria Emmelin, Malin Eriksson

**Affiliations:** 1 Department of Public Health and Clinical Medicine, Epidemiology and Global Health, Umea University, Umea, Sweden; 2 Faculty of Psychology, Ahmad Dahlan University, Yogyakarta, Indonesia; 3 Department of Clinical Sciences, Social Medicine and Global Health, Lund University, Lund, Sweden

**Keywords:** domestic violence, service agency, situational analysis, social worlds/arena map, Indonesia

## Abstract

Since the launch of a Zero Tolerance Policy in Indonesia, several policies to address domestic violence have been enacted. The obligation of local governments to establish service units for women survivors of domestic violence is one of them. Since domestic violence is a sensitive and complex issue in Indonesia it is important to understand how governmentally regulated services function in practice. This case study aimed to explore challenges faced by a local service agency in managing service provision for women survivors of domestic violence in rural Indonesia. Data from one focus group discussion (12 participants), four individual interviews, six short narratives, two days of participant observation, as well as archive reviews were collected. All data were analyzed using Grounded Theory Situational Analysis. The major challenge faced by the local agency was the low priority that was given them by the local authorities, mirrored also in low involvement by the assigned volunteers in the daily service. The study also identified a gap between the socio-cultural arena and the law & policy arena that needs to be bridged to avoid that the two arenas address domestic violence in a contradictory way. Budget allocation to support the sustainability of the daily routines of service agencies has to be given priority. There is also a need for careful considerations regarding the composition of personnel involved within daily management of service agencies addressing domestic violence. To bridge the gap between the legal systems and traditional cultural values, culturally adjusted alternative justice systems could be developed to increase women’s access to legal support.

## 1. Introduction

Violence against women was declared a global health problem by the United Nations (UN) only two decades ago trough the launching of the Declaration on the Elimination of Violence Against Women in 1993. One of the most common forms of violence against women is violence by an intimate partner within the domestic setting (Krug, Dahlberg, Mercy, Zwi, & Lozano, 2002; Heise, 2011), which has been shown to cause adverse short- and long-term physical and mental health outcomes (Campbell, 2004; Garcia-Moreno, Jansen, Ellsberg, Heise & Watts, 2005; Ellsberg, Jansen, Heise, Watts, & Garcia-Moreno, 2008). The global WHO multi-country survey, with more than 24.000 women participants, estimated the lifetime prevalence of domestic violence to range from 15% to 71% (Garcia-Moreno, Jansen, Ellsberg, Heise, & Watts, 2005).

In most countries, it has been the grass root movements that have advocated for the development of interventions and services for abused women (Appelit & Kaselitz, 2001; Heise, 2011). In Thailand (Grisurapong, 2002), and also in several European countries (Appelit & Kaselitz, 2001), domestic violence got public attention only during the last decade after feminist groups and the movement of ‘women friends for battered women’ started advocating for actions. Interventions have mainly been directed towards providing safety and practical support to abused women and their children, and also on delivering strategic support such as counseling programs, legal aid, and other empowerment programs (Schmitt & Martin, 1999). In the US, the focus has also been on legal improvements through protection orders, specialized police units and courts, as well as mandatory arrest laws (Jewkes, 2002). These services are essential, since women survivors who got support from them have perceived an improvement in their decision making capacity, an increase in their self-efficacy and coping skills, and have felt safe while in shelter (Benett, Riger, Schewe, Howard, & Wasco, 2004).

In Indonesia, a national policy called the “Zero Tolerance Policy (ZTP) on violence against women” and a national plan to eliminate violence against women was launched in the year 2000 (ZTP, MOWE, 2000). Two outcomes of these policies was the Domestic violence act in 2004 (law number 23/2004) and the enactment of a Governmental regulation (Number 4/2006) to ensure the availability of service units for women and children exposed to domestic violence, in every Province and District in Indonesia. The regulation required the local government bodies to set up certain facilities for the purpose of supporting women survivors. This included special units within the Police department, crisis centers, shelters, and making available experts and other professionals in this field. Further, it was stated that services for women survivors of domestic violence must include measures to improve physical and psychosocial health, as well as spiritual services. Since then, a number of service units run by the government and the Police Department have been established in Indonesia.

Studies examining the efficacy of service agencies within the field of domestic violence have shown some promising findings such as improvement in survivors’ safety and increased health and wellbeing after being assisted (Appelit & Kaselitz, 2001; Macy, Giattina, Sangster, Crosby, & Montijo, 2009). However, it is also known that women who experience domestic violence rarely turn to formal services for help (Garcia-Moreno, 2005), and thus there is a need for understanding the potential barriers for women to seek help from service providers. Also, there is a need to know more about how different social structures and interests might influence service provider’s possibilities to fulfill their task in offering services (Macy, Giattina, Sangster, Crosby, & Montijo, 2009).

This study aimed to explore the challenges faced by a local service agency for women survivors of domestic violence in rural Indonesia. Considering that domestic violence is a sensitive and complex issue in Indonesia it is important to understand how governmentally regulated services are being implemented and function in practice, to be able to suggest future improvements.

To date, diverse terms are used to label violence against women perpetrated by a man within a close relationship. Concepts, such as wife abuse, wife battering, and domestic violence has been replaced by intimate partner violence to avoid making assumptions about human relationships, who is involved or the direction of the violence (Kirkwood, 1993). However, the term “domestic violence” is still used in many countries, including Indonesia, and the UN bodies still refer to violence against women, when they refer to violence perpetrated by a male spouse (Garcia-Moreno, Jansen, Ellsberg, Heise, & Watts, 2005). Because “domestic violence” is the official term used in Indonesia, and because this study focuses on violence within married life, we use the terms “domestic violence”, “wife abuse” and intimate partner violence” interchangeably in this paper.

## 2. Material and Methods

### 2.1 Study Setting

We conducted the study in Purworejo District, Central Java Province, located 60 km west of Yogyakarta Province. In 2013, the Purworejo District had a population of 694,404 on an area of 1,035 km^2^. Although urban centers are found in the district, 85% of the population lives in rural areas with farming being the major occupation. In 2009, the Head of the District (*Bupati* in Indonesian language) launched an integrated service center for women and children survivors of violence (P2TP2A/Pusat Pelayanan Terpadu Perempuan dan Anak) (labelled ***the service agency*** throughout the paper). This was set up as a “non-governmental” body, coordinated by the local government, with a specific mandate to provide services that would contribute to women’s and children’s empowerment. The service agency was set up and organized by a management committee consisting of an advisory board, one full time daily officer and a group of voluntary operative persons.

### 2.2 Study Design

This study was designed as a case study. A case study approach is suitable when “how” and “why” questions are being posed qualitatively to a small number of key actors, and the focus is on contemporary underexplored events (Yin, 1994). A case study aims at bringing out the details from the viewpoints of the participants by using multiple sources of data (Tellis, 1997; Baxter & Jack, 2008). Yin (1994) identified six primary sources of data for a case study, including documentation, archival records, interviews, direct observation, participant observation, and observations of physical artifacts. Not all sources are needed in every case study, but the importance of multiple sources of data is underlined to increase the credibility of the study results (Tellis, 1997). Data from multiple sources are converged and merged in the analysis process, and treated as one piece of the “puzzle,” with each piece contributing to the researchers’ understanding of the selected case (Baxter & Jack, 2008). Data for this study were collected through focus group discussion (FGD), in-depth interviews, archival and document reviews, written narratives, and observations of the daily staffs’ activities and interaction as well as a description of the overall office setting. Within a case study design, the researcher should decide upon the main focus with regards to “the case” to be studied (Baxter & Jack, 2008). A case should be “typical, exemplary or extreme or theoretically decisive in some other way” (Ragin, 1992). Further, a case should “bring theory and practice together in a special way” (Wieviorka, 1992). In this study, *the service agency’s management and practices* was identified as the case; being a typical example of how support for women survivors of domestic violence is organized in rural Indonesia, and suitable for gaining a theoretical understanding of the challenges involved in practical implementation of governmentally regulated services in this specific setting.

### 2.3 Data Collection

The first author collected all data during a period of eight months (October 2012 – July 2013). For the FGD, we asked the coordinator of the volunteers to invite all 39 volunteers, of which twelve agreed to take part in a focused discussion about their role within the service agency. The reasons for not participating were mainly due to time constraints. For the interviews, the first author invited the coordinator of the volunteers, the daily officer and two volunteers to be interviewed about their work, and all agreed to participate. All interviews (n = 4, women) and the FGD (n = 1.11 women, and 1 man) were conducted at the service agency office during working hour, and all were tape recorded after informed consent from the participants. The interviews and the FGD lasted between 45 minutes to two hours. Participant observation was performed at the service agency office during two intensive days as well as during other data collection visits. The archival data was manually documented for further analysis. Only relevant documents available in the service agency’s office were selected for, such as the Bupati’s decree, annual reports, and annual program planning documents. In addition, the first author invited six volunteers to write short narratives about their experiences and feelings in relation to the service agency’s activities. The short narratives were written and collected either at the service agency or at the homes of the volunteers. No incentives were given to participants for their involvement in the study, but books and bulletins on women empowerment and gender based violence were donated to the service agency.

The study was approved by the District Head through a letter of approval released by the Office of the service agency in Purworejo (18/P2TP2A.Pwr/2013). Individual informed consent was asked for prior to the interviews and the writing of short narratives, to ensure the voluntary basis for participation and re-assuring the participants about the confidential handling of the information. The researcher reminded the informants of the interviews and the focus group that the data would be used for scientific purposes only, with an aim to be able to suggest improvement of the management of the service agency.

### 2.4 Data Analysis

Our analytical approach was Situational Analysis (SA), which is a development of classical Grounded Theory, as proposed by Clarke (2005). Using SA enables the researcher to focus not only *on pure “basic social process”* (Clarke, 2003, p. 553) but also on analyzing situations where power relations may exist. SA acknowledges the “field’s messiness” and the complexity in a situation of inquiry through *reflexivity*, *uncertainty*, *modesty* and *representation of contradictions* (Mathar, 2008, p. 2). The situation of inquiry in this study was the service agency’s practices in providing their service in Purworejo district.

We initiated the analysis with a manual open coding as performed in classical grounded theory, i.e. all transcripts from the interviews and the FGD, the written narratives, archives reviews, and data from the observations at the service agency office were coded. Then, the open codes were put into a Messy Situational Map (MSM). The MSM enabled us to identify all possible elements involved in our situation of inquiry. By organizing and grouping the codes into some of the basic elements of the situation as described by Clarke (2005), we then came up with an “ordered situational map” (OSM) consisting of the *major elements* in the situation of inquiry. These major elements reflected “who and what” is in the situation (Clarke & Friese, 2007, p. 272), and helped to identify what human and non-human elements that seemed to really ‘matter’. This step implied a constant comparative process of moving back and forth between data, the open codes and the identified preliminary elements, which helped to identify the most significant elements. Ten significant elements were identified when constructing the ordered OSM; Human-, non-human-, socioeconomic-, sociocultural-, management-, action/programmatic-, political-, emotional-, psychosocial-, and major debate elements. The final step in the analysis involved the development of a social worlds/arenas map. An arena is “*a field of action and interaction among a potentially wide variety of collective entities*” (Clarke, 1991 in Vasconcelos, Sen, Rosa, & Ellis, 2012, p. 123). Our social worlds/arenas map illustrated all the prominent actors involved in the daily practice of the service agency in Purworejo, as well as the different arenas they belong to. Thus, the social worlds/arenas Map further illustrated relations between the actors involved and helped to interpret the particular influence each actor has on the service agency’s performance and practices.

## 3. Results

### 3.1 The Personnel Structure of the Service Agency

The management committee of the service agency consists of in total 47 persons, divided into one advisory board (consisting of seven people), one full time officer and representative volunteers (consisting of thirty-nine people). Some of the volunteers hold a coordinating role, which implies coordinating the work of the volunteers or the work within each specific division. All the volunteers have their original affiliation at other institutions (21 at the government bodies, 14 at the civil society organizations, 4 at private institution and 2 at the Police department), which means that their responsibilities at the service agency are additional tasks or side job, but on voluntary basis.

### 3.2 The Ordered Situational Map

In total 10 elements (four sub-elements within the Human elements) were identified as significant human and non-human elements in the situation under investigation and were put into the Ordered Situational Map as described in Table 1 below:

**Table 1:** The ordered situational map of the management and practice in the service agency

Elements		Contents
	*Individual*	Bupati, the officer
		Civil servants, the representative volunteers, Teachers, Naib (KUA officers), Policy makers, Police Officers, midwives, health attendants, Politicians
**Human**	*Collective*	District Health Office, Religious Affairs Office, Police Department, Hospital and Clinics, Schools, House of Representatives, Educational Office, NGOs

	*Stakeholders*	Pregnant women, teenage girls, parents, women survivors of violence, villagers at risks, youth
	*Beneficiaries*	Pregnant women, teenage girls, parents, women survivors of violence, villagers at risks, youth
**Non-human**		Education curricula, Presidential Decree number 9/2000 on Gender mainstreaming regulation, Law number 23/2004 (Anti DV act), District Head’s decree on the formation of P2TP2A
**Socioeconomic**		Poverty, bad familial finance management, financial hardship, hunger, lack of food, jobless
**Sociocultural**		Taboo to disclose DV, women’s permit for husband’s polygamy, disempowered traditions, patriarchal, girl child marriage, better to have a status as “widow” rather than status as “belated married”, provocative dressing, lack of access for women of being heard, wife disobey, lack of awareness to report, threat toward disclosure on DV
**Management**		Monitoring and evaluation, coordination, well plan, maintain the good performance, leadership, collaboration/cooperation, irregular shift of volunteers, shelter facility, counseling room
**Action/programmatic**		Routine public lectures, dissemination, public dialogue, radio program, public lectures by Bupati, advocacy for the district regulation on free of charge medical service for women survivors, survivors’ repatriation, capacity building for P2TP2A officers, improving the minimum into maximum standard, improving the infrastructure
**Political**		No autonomy for P2TP2A, no clear fund for operational P2TP2A, bureaucracy, double/triple agencies affiliated, gender mainstreaming at the government program, district regulation, lack of commitment among the policy makers, lack of financial support from GO, huge structure of P2TP2A, fulfillment of women’s rights, shifting responsibilities, stakeholders’ commitment
**Emotional**		Happy, glad, satisfy, disappoint, dissatisfy, uncomfortable, dislike, concerned, shame, willingness, impatience, impulsivity, angry of being reported, revenge
**Psychosocial**		Dishonesty, restlessness, altruistic motive, extramarital affair, lack of openness, low tolerance toward frustration, lack of good role model, silence for the sake of harmony, egoism, attention, take the lessons learned, stubborn, lack of understanding, low commitment in marriage, weak in struggle, threat to divorce, authoritarian parenting style, narrow mindedness
**Major debate**		Full time staff VS non full time staff/representational staff; Bupati’s direct involvement at the programmatic level VS Bupati’s indirect involvement through district regulation; Disclosure about domestic violence VS silent about domestic violence; More report to the Police VS inconsistency in the Indonesia law system; Minimum standard VS maximum standard; Good network collaboration VS lack of communication within network

Overall, the above OSM lay out the major elements involved in the situation of the service agency’s management and practices. Some elements, i.e. the socio-cultural; action/programmatic; political; psychosocial; and major debate, are more salient and rich than the others, which might indicate that they ‘matter’ more within the studied situation (Clarke, 2005). The codes clustered under *psychosocial elements*, indicate a view on domestic violence as being something more related to individual characteristic than influenced by the surrounding social context. The codes grouped under *political elements* relate to the ongoing consolidation of the service agency within the bureaucracy of the local government. Further, the codes grouped as *socio-cultural elements* indicate the existence of socio-cultural values and norms embedded within the issue of domestic violence. Meanwhile, the codes clustered under *action/programmatic elements* reflect the passion embedded within this agency in doing something to address domestic violence. Finally, the codes organized under *major debate elements* reflect the tensions and dynamics involved in running this service agency.

### 3.3 Social Worlds/Arenas Map of the Service Agency’S Management

**Figure 1:**
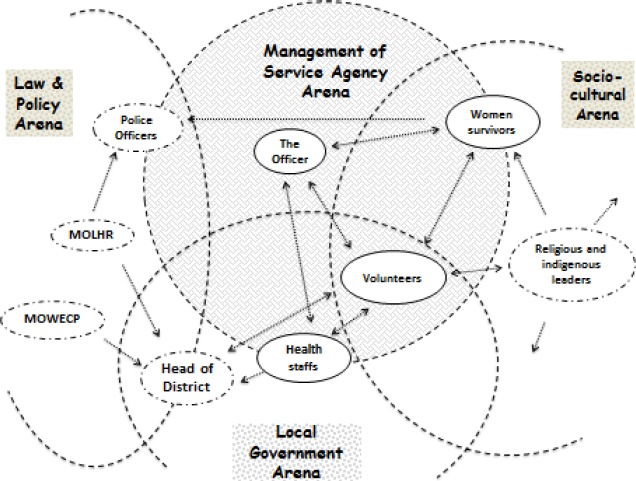
Social worlds/arenas map illustrating arenas and actors influencing the management of the service agency

The social worlds/arena’s map illustrates the most prominent actors involved and their relations, which all influence the practices and management of the service agency. Figure 1 depicts the main findings of this study, illustrated in a map of the actors involved within the arena of the service agency management. Three arenas were identified outside the main arena of the service agency management (illustrated by dotted lined big circles in the figure). These arenas represent four fields of action and include interactions of different actors in each arena. The small circles represent actors within the different arenas. Taken together, the collected data revealed eight prominent actors influencing the work and practice of the service agency. The dotted lined small circles represent actors that were not interviewed and/or observed during the data collection, while the full lined circles represent actors that were interviewed and/or observed during data collection. However, based on all data taken together, it was clear that all these actors were active and significantly influenced the work and the challenges faced by the service agency.

#### 3.3.1 The Arenas

The three arenas identified outside the main arena of *Management* *of Service Agency* were: *The Law & Policy arena*, *The Local Government arena* and *The Socio Cultural arena*. Each of these arenas has different actors, but some actors are also located within two or even three arenas. The actors that are located within the intersection of several arenas are key actors within the situation of inquiry, as their discourses are owned by several arenas. The district head, the volunteers, the officer and women survivors are central for the work and practices of the service agency. The arrows in the figure indicate interaction, influence, or negotiation between actors in each arena.

The management arena has an overlap with the local government arena since the formation of the service agency was based on the local government’s decrees. In addition, the management committee’s personnel, i.e. the volunteers, mainly come from the government bodies. The overlap between the management arena and the socio cultural arena is bigger compared to the overlap between the management and law & policy arenas. This illustrates a larger space of interaction between the Management and the Socio-cultural arenas within the work of the service agency. Further, the gap (no overlap) between the socio-cultural arena and the law & policy arena indicates no interaction between these two arenas.

#### 3.3.2 The Actors’ Different Contributions to the Management and Practices of the Service Agency

The Ministries - MOWECP and MOLHR: *“Present but relying on others’ actions”*

Two ministries were identified as significant actors within the law and policy arena. The Ministry of Law and Human Rights (MOLHR) was the main actor responsible for the enactment of Domestic Violence Act and the establishment of service units for women and children exposed to domestic violence. The Ministry of Women Empowerment and Child Protection (MOWECP) has been the main collaborator of the Ministry of Law and Human Rights in implementing those two regulations and these ministries have their representatives in the district, which potentially could facilitate the implementation of regulations at district level. However, the results show that in practice, the ministries do very little at district level, but rely on other actors to act. Thus, they influence the local actors such as the Bupati and the police officers, since their work is regulated by those ministries.

The Police Officers: *“mandated but reactive”*

The police department is located in the intersection of the law and policy arena and the management arena. The police department has their representatives at the service agency’s; one in the advisory board, and two at the representative volunteer level. The involvement of the police department is mandated from the ministry to fully support the service agency in addressing domestic violence, illustrated in Figure 1 by the arrow going from the Ministry to the police officers. However, their direct involvement is very dependent on women reporting to them. The Indonesian Law states that the women themselves should make the legal report and could not be represented by the family or other persons, and the police should follow up the women’s report for further action upon their assessment to the women’s situation. In short, the police can do nothing to protect the women survivors, unless the women come and report their abused husband/partner. In the figure, this is illustrated by the arrow going from women survivors to the police department.

The Bupati: *“Powerful but passive”*

The Bupati belongs both to the law and policy arena as well as to the local government arena. As one of the members of the advisory board at the service agency, the Bupati has a crucial role in providing guidance to the management committee in operating the service. However, the involvement of the Bupati on the groundwork of the service agency was considered as low.

*“I do hope that the Bupati could bring this issue with him, delivered everywhere, every time he does his public duties. This is also his duty, right? If he does, it will have a different effect for those who listen”* (female volunteer)

Another situation faced by the agency was lack in getting financial support to operationalize the services from the government, which of course was referred to the Bupati’s desk.

*“We only got a few starting funds, during the initial launching of this office. Within the next year from that, we got nothing, and I endorsed my own way in finding any support for this office, so I could take care for the survivors. I negotiated many times about this to the structural body and the Bupati himself, but… Well, let’s hope this year (2013) we will get it”* (female volunteer).

The Bupati is influenced by the Ministries as being regulated to set up the service (illustrated in the figure by the arrows from the Ministries to the district head), and then, he influences the volunteers - as well as the advisory board - by being the one assigning them (illustrated in the figure by the arrow from him to the volunteers). Despite being a powerful person in the district, and the representative body of the state he was perceived as less committed and quite passive in the work of the service agency.

The Health Staff: *“Concerned but limited by profession”*

The health staff belong both to the management arena and the local government arena, since their engagement with the service agency is regulated by law. There were two representatives from the government hospital assigned at the management committee of the service center. They saw a need of a separated ward at the hospital to give better care and privacy for the women. However, given that their workplace is in the hospital, they have no power and capacity to realize these ideas.

*“I think the current decree is not enough, since it does not cover the medication fee waiver for women survivors. We also still could not protect their privacy if they stay for being hospitalized due to violence. They deserve to have that specialized treatment. Until today, we can only perform in a very limited facility…we wish we could do better…but you know…” (male volunteer)*.

The arrow going between the health staff and the volunteers illustrates a mutual influence through referrals going both directions, while the arrow going from the health staff to the District head illustrates their attempts to influence decisions by the Bupati.

The Officer: *“Central but powerless”*

The daily officer plays a central role for the service agency and thus is placed at the center of Figure 1 within the management of service agency arena. The daily routines of the service agency were handled by one female administrative officer that was paid by the committee management for her full time availability at the agency. She was the responsible person for the internal communication between the volunteers, external liaison between institutions in terms of administrative and communication, and between survivors and the representative volunteers for further referral. However, since her tasks are technical and administrative, she has no authority to make any decision regarding the management of this service agency.

*“All decisions related to survivors, networking, and other institutional matters are in the hand of the coordinator of the volunteers and I manage only for administrative things” (the officer)*.

The arrows in Figure 1 thus illustrate a mutual influence between the officer and the volunteers, the health staff and the women survivors.

The Volunteers: *“Assigned but not able to prioritize”*

The volunteers belong to the management arena and the local governmental arena since their existence are formed and regulated by the local government. In addition, they are also located within the socio-cultural arena since their actions are very much influenced by religion as well as by local traditions and beliefs. Participation of all volunteers in this service agency was an additional task for them. Their standby shifts at the agency was arranged based on their availability of time in-between their ordinary working hours. Only few volunteers were able to spend their time more consistently at the service agency office since their main working desk was located next to the service agency. The observations in the office revealed a quiet atmosphere during the working days. Most of the time, only the full time officer was present and not all volunteers came for their assigned standby shifts. The inconsistent attendance of many of the volunteers in fulfilling their stand by shifts was a source of frustration by others and raised a feeling of unfairness’.

“This agency should be supported by all of the volunteer, not only us…few of us that most of the time sitting in here…all should have the same awareness to overcome this problem, the same mindset…not only think about their own work…” (female volunteer)

The volunteers are the “heart” of the agency, though with the current practice for assigning the volunteers, it is impossible to achieve an optimal result since their voluntary work and attendance at the agency cannot be prioritized.

The Women Survivors: *“motivated but lack cultural support”*

The women survivors are located at the border of two arenas; the management service agency arena and the socio-cultural arena. Their needs are central for the service agency, and thus there is a mutual influence between the women and the officer, the volunteers and the health staff (as illustrated by the arrows). However, the women in need of the service are also very much influenced by local traditions and beliefs (illustrated by the arrow going from religious and indigenous leaders to the women). Domestic violence is culturally a very sensitive issue to be discussed in the public sphere, which means that it is not an easy thing to reveal by the local women who are exposed to it.

“Usually women come with their parents and make a formal report, and we discuss further for exploring the follow-up for her. It must be hard for the women if they have to come alone, and report their own problem, since this problem needs to be decided…will be inviting the husband for further counseling or what?” (female volunteer)

Despite the existence of the Anti DV law and other national policies it seems that women, on their own, will not access service without encouragement from significant social supporters such as family member and friends.

The Religious and Indigenous leaders: “*powerful and influential but invisible*”

This actor was not directly visible within the socio-cultural arena, but become visible through other actors that referred to these leaders way of thinking when addressing domestic violence. Prioritizing and spreading the message of the importance of “family unity” is one of the most dominating norms within this site, steered by religious interpretations. The norm of “the man as the leader and head of the family household”, as taught by the religious leader, is almost unconditionally confirmed in the society. Another norm widely supported in this society is that “divorce is an act that wraths God”. The official brochure of the service agency has a picture of a broken heart at the cover, with the title underneath “*Do not let domestic violence break our family unity*”. The preference of sidestepping the legal and justice system when working with domestic violence has become the mainstream idea among the volunteers. This means that the service agency, to a high extent, conforms to the cultural traditions of avoiding sanctions (impunity) for abuse within marriage.

#### 3.3.3 The Gap Between the Law and Policy Arena and the Socio-Cultural Arena

Figure 1 shows agap between the law and policy arena and the socio-cultural arena. The law and policy arena contains different actors connected to the legal and justice system and the structure of law. The President of Indonesia (Megawati) enacted the anti-domestic violence law in 2004, and also assigned different ministries to ensure it’s implementation. The Ministries (MOLHR and The MOWECP) and the police officers have become the salient actors belonging to this law and policy arena. Meanwhile, the socio-cultural arena upholds the local traditions and beliefs that somewhat contradict the existing laws and regulations. These two different arenas are opposite to each other, and a huge challenge faced by the service agency is how to ensure women survivors’ access to the law and policy arena and get the maximum benefit from it. One of the volunteers at the service agency expressed her concern regarding this particular issue:

*“Our community is still viewing domestic violence as something taboo to be disclosed to others. And if they want to solve that problem, we suggest them to solve through kinship discussion first, and not to report it to the Police… we strongly suggest not to report it legally”* (female volunteer).

## 4. Discussion

Overall this study captured several challenges faced by the service management in providing services to address domestic violence in the Purworejo district. We observed a gap between the socio-cultural arena and the law and policy arena that needs to be bridged by either the service agency management or the local government to avoid that the two arenas address domestic violence in a contradictory way.

### 4.1 Bridging the Gap Between the Legal System and the Socio-Cultural Practices

A previous study in this setting found that one of the risk factors for women to be exposed to domestic violence was their traditional gender norms (Hayati, Högberg, Hakimi, Ellsberg, & Emmelin, 2011). Our present study found that avoidance to disclose domestic violence experience to outsiders is still preferred within this society. Also, the value of “family unity” is highly prioritized. This indicates the presence of social and cultural norms that may accept violence against women (Heise, 2011). Thus, criminalizing domestic violence (Fagan, 1996), in this setting implies confronting and challenging existing cultural values and norms. Other studies have shown that cultural norms are difficult to challenge when addressing domestic violence in developing countries (Bott, Morrison, & Ellsberg, 2005; Heise, 2011). Many women tend to refuse accessing the legal system to avoid punishing or sending their abusive partners to jail. However, it has also been shown that the presence of legal and justice services is crucial for women to overcome a life-threatening situation due to abuse (Heise, 2011).

To decrease the gap between the modern legal system and traditional cultural values some grass root initiatives have focused on developing alternative justice procedures (Heise, 2011). Restorative justice is an initiative to enlarge the circle of people involved in the criminal justice case by including also victims and community members (Zehr, 2002). An example of restorative justice from South Africa is the development of a victim offender conference (VOC) model, where women survivors, the abuser, the family and the community are joining in a mediation process. The idea is that it is important for all parties to understand the violence that has occurred within a relationship. This is seen as important for understanding the causes of the violence, as well as for what is needed of the abuser to take his responsibility as a perpetrator (Dissel & Ngubeni, 2003). In our study setting, where women still get very little support to report domestic violence for legal action (especially if there is a risk of a jail sentence) this model could offer an alternative for seeking justice for women survivors. However, using restorative justice might also put women at a risk of being recommended to maintain the family harmony and stay in an abusive relationship, given that the women live in a culture where traditional gender roles exist (Heise, 2011).

### 4.2 Improving the Organization and Coordination of the Service Agency

This study shows that the organization of the local service agency, despite the good will of all involved actors, was not optimal. The dependency of voluntary work creates an arbitrary organization and signifies that the work of the service agency is not a significant public priority. The lack of engagement from the responsible governmental representatives (The Ministries and the District Head) further signals that the work of the service agency is not prioritized in the District. Using the policy implementation model from Matland (1995), the formation of this service agency management can be said to reflect a “top down” model of policy implementation. A top down model implies implementing a policy in an authoritative way, seeing the policy mainly as an administrative process and ignoring the political and practical aspects of its implementation. In our case study an example is the lack of commitment of the Bupati to allocate an annual budget for the agency despite a decree about this being an obligation. According to the Indonesian DV Act and the Ministry regulation, the district government has no bargaining position to refuse to set up a service provider even though they have no resources or plans on how to take on the responsibilities it entails to fully implement that policy. A “bottom up” model would put more priority on involving the implementing agencies and the target population in the planning and designing of the services (Matland, 1995).

The service agency in this study was a coalition between different agencies and can be described as a form of coordinated community approach, a combination of a Task Force and a Community Partnering model (Hart, 1995). A Task force model is based on coordinating all components of the criminal justice system to address domestic violence, while a Community Partnering model is based on coordinating a working group with specific resources and tasks and with members representing different types of expertise. However, our results indicate that a Task Force model needs to acknowledge the potential cultural barriers for approaching the legal system. Also, in another study, a coordinated community response to domestic violence was found to lack coordination across the networks involved (Allen, 2006). This means that a Community Partnering model needs to take into account the prerequisites needed for the involved expertise to be able to prioritize and coordinate their work.

Even though this study was not designed to evaluate the work of the service agency, the results clearly indicate that organizing the work at the service agency through volunteers obstructs both availability and performance. Most serious is however that the agency’s practices have not yet been able to facilitate the women survivors’ access the judicial system and achieving the benefits of the anti-domestic violence Act.

### 4.3 Methodological Considerations

This study was conducted using a case study design, implying the use of multiple methods for data collection. The use of multiple data sources increased the credibility of the results since the research question can be captured from different angles (Creswell, 2007). Further, triangulation in investigators/researchers (with different disciplinary backgrounds) in the analysis and interpretation further strengthened the credibility of the study results. The first author’s prolonged engagement with the study area and the personnel at the service agency further ensured interaction and trust between the researcher and the informants also contributing to the credibility of the results (Dahlgren, Emmelin, & Winkvist, 2004).

However, a limitation of the study is that the interviews, the FGD and the observations mainly involved those volunteers that were more frequently on shift in the office. Thus, we cannot be sure that we have captured the full variation in experiences and perceptions of those working at the service agency.

Qualitative research aims for theoretical generalizations (Dahlgren et al., 2004) but the transferability of results to other geographical contexts is to be judged by others. However, since the implementation of the Governmental regulation to set up service units for women and children survivors of violence is similar in all parts of Indonesia, we believe that the challenges faced by this specific service agency, might be valid also for other service units in Indonesia. Further, the identified gap between the legal system and the socio-cultural practices in addressing domestic violence is most likely to be valid also in similar socio-cultural settings.

## 5. Conclusions and Policy Implications

Based on the findings from this study we have identified a need to improve the practices of local services to address domestic violence, in this particular study setting but most probably throughout Indonesia and in other similar socio-cultural settings. The implications of this study suggest that:

- Careful considerations are needed regarding the composition of personnel involved within daily management of service agencies. A small solid and full-time employed team with comprehensive knowledge on law and policy, gender and its relation to domestic violence and with adequate skills in interviewing, documenting, referring and public speaking, will constitute a more solid base for the work on addressing domestic violence, compared to a model based on voluntary work.
- Advocating for budget allocation to support the sustainability of the daily routines of service agencies should be made a priority for the political leadership in its annual budget plan.
- Culturally adjusted alternative justice systems might be needed to bridge the gap between the legal system and traditional cultural values. Involving not only the victim and the perpetrator in the judicial process, but also locally trusted leaders is one way to decrease the existing gap.
- Public dissemination of information and education on the concept of gender, gender based violence, and the existing laws and policies related to domestic violence are needed. Using the local traditional approaches and involving indigenous and/or religious leaders would most likely increase the impact of such activities.
